# Nivolumab-induced Third Degree Atrioventricular Block in a Patient with Stage IV Squamous Cell Lung Carcinoma

**DOI:** 10.7759/cureus.4869

**Published:** 2019-06-10

**Authors:** Hazim S Bukamur, Haitem Mezughi, Emhemmid Karem, Ibrahim Shahoub, Yousef Shweihat

**Affiliations:** 1 Internal Medicine, Marshall University, Joan C. Edwards School of Medicine, Huntington, USA; 2 Pulmonology, Marshall University, Joan C. Edwards School of Medicine, Huntington, USA; 3 Internal Medicine, University of Kentucky College of Medicine, Lexington, USA

**Keywords:** check-point immune inhibitor, nivolumab, rare drug-induced complication, autoimmune-induced myocarditis, third degree atrioventricular block.

## Abstract

In the era of immune checkpoint inhibitors, pulmonary and critical care physicians frequently encounter patients taking these medications, usually after being admitted to the intensive care unit with life-threatening complications. These complications are rare, present with nonspecific and vague symptoms, which may delay the treatment and have high mortality. We report a very rare complication, with only two previously reported cases of a severe and potentially fatal side effect associated with anti-programmed cell death protein 1 (PD-1) immunotherapy with nivolumab. We provide a literature review to increase physicians’ awareness about this rare side effect and suggest some recommendations derived from our experience.

## Introduction

Recently, immune checkpoint inhibitors have transformed cancer therapy and are recognized as a novel treatment option for patients with advanced cancer stages [1-2]. Nivolumab is an immunoglobulin G4 monoclonal antagonist antibody to programmed cell death protein 1 [3-4]. It was recently approved as a standard treatment for patients with advanced, previously treated squamous-cell carcinoma of the lung [2-5]. It has been associated with severe and fatal immune-related adverse events [5]. One of such events is myocarditis, which can impair the conduction of cardiac electrical stimuli, leading to the development of a third-degree atrioventricular block with bradycardia and fatal hemodynamic consequences [6-7]. Here, we report a very rare complication, with only two previously reported cases of a severe and potentially fatal side effect associated with anti-programmed cell death protein 1 (PD-1) immunotherapy with nivolumab.

## Case presentation

**Figure 1:**
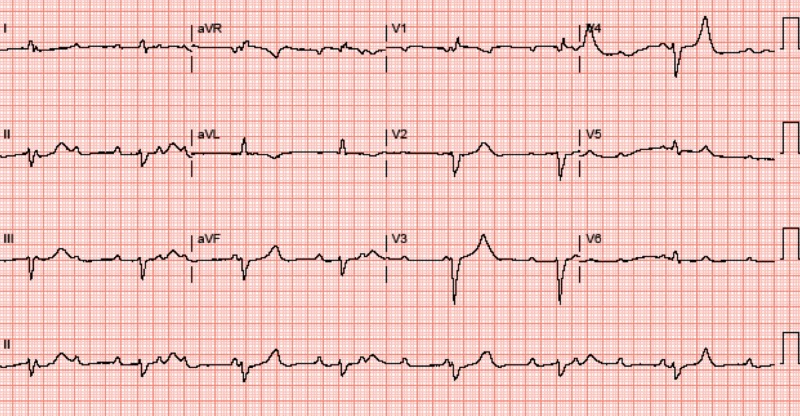
Electrocardiogram showing complete atrioventricular block

An 88-year-old female patient with a past medical history of hypertension and hyperlipidemia on a statin was recently diagnosed with stage IV squamous cell carcinoma of the lung. Treatment was initiated with paclitaxel which she did not tolerate because of the side effects and, therefore she was transitioned to nivolumab. After completing two cycles of nivolumab, 240 mg every two weeks, she was admitted with muscle aches and proximal weakness. Laboratory analysis showed total creatinine kinase (CK) of 4050 U/L, myoglobin 5373 µg/L, troponin 2.2 ng/mL, aspartate aminotransferase (AST) 308 units/L, alanine aminotransferase (ALT) 206 units/L, thyroid-stimulating hormone (TSH) 4.480 mIU/L, and free thyroxine (T4) 1.25 pmol/L. The statin was stopped, and the patient was started on high-dose pulse steroids. The echocardiogram showed increased myocardial thickness with no signs of ischemia. During her hospital stay, she developed sinus bradycardia that progressed to complete atrioventricular block as seen in the electrocardiogram (ECG) (Figure 1).

An electrophysiology specialist was consulted, and a temporary transvenous pacemaker was inserted, followed by a permanent pacemaker. The patient’s overall condition improved and she was discharged to a skilled nursing facility.

## Discussion

An extensive literature review identified two reported cases of complete heart block associated with the use of nivolumab. The first case was described by Johnson et al. The patient was initially administered with a combination of ipilimumab with nivolumab for metastatic melanoma and presented with atypical chest pain, shortness of breath and fatigue. The initial laboratory analysis showed significantly elevated total CK, CK-MB, and troponin. The ECG showed a prolonged PR interval with normal QRS. Within 24 hours, the patient developed a complete heart block, and myocardial biopsy revealed myocarditis. Treatment commenced with high-dose glucocorticoids (intravenous methylprednisolone administered at 1 mg/kg per day) within 24 hours after admission. This particular patient later died from multisystem organ failure and refractory ventricular tachycardia. The patient was not on a statin, and the only possible risk factor was hypertension [6]. 

The second case was reported by Behling et al. This was a male patient with metastatic melanoma who was started on nivolumab monotherapy and had been previously treated with sorafenib which was stopped due to corneal ulceration. Additional medications of importance used by the patient included a statin. This male patient presented to the emergency department with worsening dyspnea, dysphagia and muscle pain. The laboratory analysis showed significantly elevated CK, myoglobin, and troponin. However, a myocardial biopsy was not performed. Additionally, the patient had positive alanyl-tRNA synthetase antibodies (PL-12-B) and anti-signal recognition particle (SRP-B) antibodies. Treatment commenced with intravenous prednisone (1.5 mg/kg body weight). This particular patient died from hemodynamic instability, acute respiratory failure, and pneumonia [7]. According to the authors, there was a delay in the management of this patient because his initial symptoms were thought to be secondary to an underlying disease (chronic obstructive lung disease) and statin use, which was stopped. In addition, the rarity of these complications makes it even harder to diagnose [7]. 

In our case, nivolumab was started for stage IV squamous cell carcinoma. The patient presented with myositis, myocarditis, hepatitis, and thyroiditis that was attributable to nivolumab. While in the hospital, the patient developed a complete heart block and was managed by a temporary pacemaker followed by a permanent one. High-dose pulse steroids were started on admission for the immune-related adverse events. A collaborative, multidisciplinary approach by an intensivist, gastroenterologist, endocrinologist, cardiologist, and oncologist with early involvement in patient care, as well as an awareness of this rare side effect based on the previously reported cases, resulted in early diagnosis and treatment of this complication with a favorable outcome. 

Patients with myocarditis secondary to nivolumab can present with dyspnea, chest pain, lower limb swelling, and neck vein congestion [1,8]. Patients may also present with fatigue, muscle pain, and dysphagia secondary to concomitant myositis [6-7]. The average time from starting nivolumab to exhibiting these complications is 4-8 weeks [6-9]. Patients could have vague and nonspecific symptoms with a wide range of differential diagnosis, which may elude the practitioner and delay the diagnosis [6]. Especially, these medications are new, used mainly by oncologists, and these complications are very rare [9] and not encountered too often by intensivists and other specialties on a regular basis. Thus, critical care and primary physicians should be aware of these complications to encourage early diagnosis and management for better outcomes. 

Laboratory workup may reveal elevated CK, myoglobin, and troponin [1,6-7]. A recent retrospective study reported that 94% of myocarditis cases had a troponin elevation [9] and it was found to be a useful tool to predict adverse cardiac outcomes. Patients may demonstrate seroconversion after treatment with nivolumab with positive extractable nuclear antigen screen, positive PL-12 and SRP-B antibodies [2]. The EKG could be normal, but it may show a complete heart block with bradycardia [1,7]. Ischemia should be ruled out by serial troponin, echocardiogram, and occasionally left heart catheterization (LHC). Transthoracic echocardiogram (TTE) could be normal or it may show thick myocardium, related to myocardic edema [7]. The TTE could also facilitate the estimation of the ejection fraction and the evaluation of other causes. Physicians should not rely only on left ventricular ejection fraction (LVEF) to determine the severity of nivolumab related myocarditis, as it has been reported in one study that 38% of patients with immune check point inhibitors (ICI) related fulminant myocarditis had normal LVEF [9]. A cardiac magnetic resonance imaging (MRI) scan has been used to diagnose myocarditis by demonstrating late gadolinium enhancement [9], edema and hyperemia [10]. 

The gold standard for diagnosing myocarditis is myocardial biopsy [10], which may demonstrate an intense, patchy lymphocytic infiltrate within the myocardium [9], that may also involve the cardiac sinus and atrioventricular nodes, positive for the T-cell marker CD3 or the macrophage marker CD68 [1,10-11]. 

Current guidelines recommend a baseline workup of ECG, to consider troponin and upon signs or symptoms of myocarditis to get an ECG, troponin, brain natriuretic peptide, echocardiogram and chest X-ray. May consider additional testing after an early consultation with a cardiologist, which may include stress test, cardiac catheterization, and cardiac MRI. The recommended management of myocarditis is to immediately hold nivolumab and permanently discontinue after grade 1, to start high dose corticosteroids (oral or IV depending on symptoms). May offer early institution of cardiac transplant rejection doses of corticosteroids (methylprednisolone 1 g every day) or escalation to other immunosuppressive drugs (such as infliximab, mycophenolate mofetil and antithymocyte globulin ATG) to be considered if symptoms did not respond to steroids [12-13]. 

Physicians need to be aware of this rare condition and to have a high index of suspicion because an early diagnosis improves the outcome [12-13]. We suggest the use of an algorithm on patients who will be started on nivolumab and present in the future with these complications. First, before starting the medication, a thorough history and physical exam is needed with an evaluation of risk factors for myocarditis and heart disease, any personal history of autoimmune disease, diabetes, hypertension, and hyperlipidemia [6-7]. It has been reported that patients who have ICI related myocarditis had a higher prevalence of diabetes mellitus and sleep apnea, and a higher body mass index in comparison to controls [9]. The patient’s medication list should be thoroughly evaluated, and any medication that could cause or augment these side effects such as statins should be discontinued. However, this option should be left to the physician discretion and patient choice, after considering the patient’s risk factors. Physicians may also consider checking troponin level at base line and at each cycle as the median time to myocarditis is 4-8 weeks, which is after the second dose of ICI [9]. Patients who present with dyspnea, chest pain, muscle aches, or generalized weakness should be evaluated thoroughly using CK, myoglobin, Troponin I, ECG, and TTE [6-7] to rule out ischemic heart disease and evaluate for myocarditis. For those patients with suspected myocarditis, the gold standard for diagnosis is myocardial biopsy [6,10]; however, cardiac MRI and or positive typical antibodies may preclude invasive procedure, especially in critically ill patients [7].

## Conclusions

Myocarditis with or without a complete heart block is a life threatening complication of nivolumab. Patients on nivolumab monotherapy or in combination with other agents, should be monitored closely for such serious side effects. From our experience and the other two cases mentioned above, we recommend early admission of unstable patients to the intensive care unit with a collaborative, multidisciplinary approach. While our patient has survived, the patients in the other two reported cases did not, indicating the seriousness of these complications. If a patient develops complete heart block, a temporary pacemaker should be inserted followed by a permanent one. In addition, physicians need to be aware of these rare complications, especially with the expanding indication for nivolumab, which could increase the prevalence of these complications.
